# Sex Differences in the Prevalence, Awareness, Treatment, and Control of Diabetes Mellitus Among Adults Aged 45 Years and Older in Rural Areas of Northern China: A Cross-Sectional, Population-Based Study

**DOI:** 10.3389/fendo.2019.00147

**Published:** 2019-03-14

**Authors:** Jingyan Li, Jingxian Ni, Yanan Wu, Hongyan Zhang, Jie Liu, Jun Tu, Jingqiu Cui, Xianjia Ning, Jinghua Wang

**Affiliations:** ^1^Department of Endocrinology and Metabolism, Tianjin Medical University General Hospital, Tianjin, China; ^2^Department of Neurology, Tianjin Medical University General Hospital, Tianjin, China; ^3^Department of Epidemiology, Tianjin Neurological Institute, Tianjin, China; ^4^Key Laboratory of Post-Neuroinjury Neuro-Repair and Regeneration in Central Nervous System, Tianjin Neurological Institute, Ministry of Education, Tianjin, China

**Keywords:** sex differences, diabetes mellitus, prevalence, awareness, treatment, control

## Abstract

**Aims:** Diabetes mellitus (DM) has reached epidemic proportions among adults worldwide, with China having the world's largest population of individuals with the disease. Although the consequences of low rates of awareness, treatment, and control of DM are understood, sex-related differences in these rates remain unknown. We assessed sex-related differences in the prevalence, awareness, treatment, and control of DM in a low-income, rural population in China.

**Materials and Methods:** Individuals ≥45 years old without cardiovascular disease were recruited into this study. The prevalence, awareness, treatment, and control of DM in both men and women were assessed after accounting for age, educational level, body mass index, and blood pressure.

**Results:** A total of 3,725 participants (women, 58.8%) were included. A male preponderance in the prevalence of DM was found among individuals aged 45–54 years, whereas there was a female preponderance among patients aged 65–74 years and among those who were illiterate. Among individuals with >6 years of formal education, overweight individuals, and normotensive individuals, there was greater DM awareness among women than among men. There was also a higher DM treatment rate among overweight women than among overweight men. However, better disease control was observed among men than among women for individuals aged 55–64-years, those with 1–6 years of education, and those with stage II hypertension.

**Conclusions:** These results suggest that DM awareness should be improved among men and that regular DM screening should be implemented for men, especially young men. In addition, disease education and management should be strengthened for elderly women, especially those with low levels of education. Further studies are necessary to explore this situation among a representative population sample in China in order to establish a valid protocol against DM.

## Introduction

DM is an important worldwide public health issue (1, 2). In fact, the International Diabetes Federation (IDF) predicts that the number of people with DM will increase from 240 million in 2007 to 380 million by 2025 and will further increase to 439 million by 2030 (3, 4). In 2013, China already had the world's largest population of individuals with DM (5), and the number of people with DM is estimated to increase to 42.3 million by 2030 (6). In addition to the human burden, diabetes also causes a huge financial burden. According to the IDF, 13% of China's 2010 health expenditure (USD $25 billion) was attributed to diabetes management (7). These findings highlight the grim situation of the diabetes epidemic in China.

Many complications, such as diabetic retinopathy and diabetic nephropathy, are associated with DM and may lead to blindness and kidney failure (8, 9). The disease is also a major risk factor for ischemic heart disease and stroke, causing approximately 1.29 million deaths worldwide in 2010 (10, 11). Studies have shown that the consequences of DM complications can be alleviated through proper patient management and education (12). However, almost half of DM patients, worldwide, have not been diagnosed; in China, an estimated 70% of individuals with diabetes have not been diagnosed (13, 14). In the United States, the prevalence of DM among elderly individuals is 21%, and the awareness, treatment, and control rates are 71, 51, and 50%, respectively (15). However, in China, DM prevalence and rates of awareness, treatment, and control are relatively low, at 6.4, 45.8, 42.5, and 20.8%, respectively (16).

Many studies have examined DM prevalence as well as the awareness, treatment, and control rates, globally. To the best of our knowledge, however, few studies have investigated the potential sex-related differences in these rates (17). Moreover, China remains a largely agricultural country, with residents registered as rural dwellers accounting for approximately half of the Chinese population. There is considerable disparity in the socioeconomic statuses and lifestyles for people in these areas compared to those in urban Chinese or Western populations, especially between men and women. Despite the paucity of studies examining sex-related differences in the awareness of DM in rural China, a high prevalence of hypertension and incident strokes have been reported in the population (18–21). Thus, we aimed to assess the sex-related differences in DM prevalence and its awareness, treatment, and control among low-income adults, ≥45-years-old, in rural areas of northern China.

## Materials and Methods

### Participants

This population-based, cross-sectional study was performed between April 2014 and January 2015. In brief, the study population comprised 14,251 participants from 18 administrative villages in rural Tianjin, China. About 95% of the participants were low-income farmers (2014 per capita disposable annual income of < $1600 US) (22). All residents, aged ≥45 years and without cardiovascular disease (CVD), were recruited to participate.

The previously described study design was approved by the ethics committee for medical research at Tianjin Medical University General Hospital (23–25). Further, the study methods were conducted according to the approved guidelines, and informed consent was obtained from all participants.

### Information Gathering

Demographic information, including name, sex, date of birth, and educational level, were obtained from existing records. All other data were obtained by trained epidemiology researchers, through face-to-face interviews, using a specifically designed questionnaire. The participants were categorized into four age groups: 45–54 years, 55–64 years, 65–74 years, and ≥75 years. Educational levels were divided into three groups according to the number of years of formal education: illiterate (no formal education), 1–6 years, and >6 years.

Individual and family medical histories were obtained through patient self-reports or from medical records and included evidence of hypertension, DM, stroke, transient ischemic attack, and coronary heart disease. Lifestyle characteristics included cigarette smoking and alcohol consumption. Cigarette smoking was defined as smoking >1 cigarette/day for at least 1 year; participants were categorized as never smokers, ever smokers (ceased smoking for ≥6 months), and current smokers. Alcohol consumption was defined as drinking more than 500 g of alcohol/week for ≥1 year; participants were categorized as never consumed alcohol, ever consumed alcohol (temperance for ≥6 months), and current alcohol consumption groups.

### Measurements

Physical examinations included blood pressure (BP, including systolic [SBP] and diastolic [DBP] BP), height, and weight measurements performed at the local village clinic during the baseline survey. Body mass index (BMI) was calculated as the person's weight (kg) divided by the square of his or her height (m^2^). The level of fasting plasma glucose (FPG) was measured at the Ji County People's Hospital.

### Definitions

Hypertension was defined as SBP ≥140 mmHg, DBP ≥90 mmHg, or as the existence of the requirement of medications for hypertension. DM was defined as FPG level ≥7.0 mmol/L, a prior history of diagnosed diabetes, or requirement of insulin or oral antidiabetic drugs in the patient (26). According to their BMI, individuals were classified as obese (BMI ≥28.0 kg/m^2^), overweight (BMI of 24.0–27.9 kg/m^2^), or of normal weight (<24.0 kg/m^2^) (27).

DM awareness was defined as the self-reporting of any prior DM diagnosis made by a physician. Treatment of DM was defined as using at least 1 prescription medication for the treatment of DM within the previous 2 weeks among those aware of their DM. Controlled DM was defined as an FPG level < 7.0 mmol/L among DM patients receiving treatment.

### Statistical Analyses

Continuous variables are presented as means with standard deviations and were compared between two groups using Student's *t*-tests. Categorical variables are presented as numbers with frequencies and were compared using chi-squared tests. Multiple linear regression analyses were used to evaluate the associations of high DM prevalence within a group with those factors showing statistical significance in the univariate analysis. The relationships are presented as odds ratios (ORs) and 95% confidence intervals (CIs). SPSS for Windows (version 19.0; SPSS, Chicago, IL, USA) was used for analyses; *P*-values < 0.05 in the two-tailed tests were considered statistically significant.

## Results

The selection process for participants has been described previously (23). Briefly, 4012 individuals were interviewed, during the study period, from among 5380 qualified residents, yielding a response rate of 75%. Finally, 3725 subjects were enrolled in the study, after excluding 223 residents with histories of CVD or stroke and 64 subjects without FPG level measurements.

### Demographic Characteristics

Of the 3725 included participants, 58.8% were women. The mean age of the participants was 59.96 years, but those in the DM group were older (61.8 years) than those in the non-DM group (59.7 years; *P* < 0.001). Among the participants, >60% had ≤ 6 years of formal education, 68.4% had hypertension, and 65.8% were overweight or obese. Participants with DM were more likely to be less well educated and have higher SBP and DBP measurements than were those without DM (Table 1).

**Table 1:** Demographical Characteristics for all participants in this study by Gender.

**Groups**	**Total**	**DM**	**Non-DM**	***P***
Gender, *n* (%)	3,725 (100)	533 (100)	3,192 (100)	0.719
Men	1,536 (41.2)	216 (40.5)	1,320 (41.4)	
Women	2,189 (58.8)	317 (59.5)	1,872 (58.6)	
Age, means (SD), years	59.96 (9.68)	61.80 (9.46)	59.65 (9.68)	<0.001
Age group, *n* (%)				<0.001
45~54 years	1,204 (32.3)	129 (24.2)	1,075 (33.7)	
55~64 years	1,495 (40.1)	218 (40.9)	1,277 (40.0)	
65~74 years	716 (19.2)	136 (25.5)	580 (18.2)	
≥75 years	310 (8.3)	50 (9.4)	260 (8.4)	
Education, means (SD), years	5.48 (3.54)	4.98 (3.61)	5.56 (3.52)	<0.001
Education, *n* (%)				<0.001
0 years	650 (17.4)	113 (21.2)	537 (16.8)	
1~6 years	1,666 (44.7)	253 (47.5)	1,413 (44.3)	
> 6 years	1,409 (37.8)	167 (31.3)	1,242 (38.9)	
SBP, means (SD), mmHg	146.51 (22.11)	153.45 (21.74)	145.35 (21.96)	<0.001
DBP, means (SD), mmHg	86.84 (11.37)	87.91 (10.60)	86.66 (11.49)	0.013
Hypertension, *n* (%)				<0.001
Yes	2,549 (68.4)	445 (83.5)	2,104 (65.9)	
No	1,176 (31.6)	88 (16.5)	1,088 (34.1)	
Blood pressure level group, *n* (%)				<0.001
Normal BP	1,176 (33.3)	88 (17.6)	1,088 (35.8)	
Stage I hypertension	1,264 (35.8)	198 (39.7)	1,066 (35.1)	
Stage II hypertension	831 (23.5)	164 (32.9)	667 (22.0)	
Stage III hypertension	263 (7.4)	49 (9.8)	214 (7.1)	
BMI, means (SD), years				<0.001
BMI group, *n* (%)	25.57 (3.68)	26.81 (3.74)	25.36 (3.62)	<0.001
Normal weight	1,275 (34.2)	111 (20.8)	1,164 (36.5)	
Over-weight	1,572 (42.2)	246 (46.2)	1,326 (41.5)	
Obesity	878 (23.6)	176 (33.0)	702 (22.0)	
Smoking status, *n* (%)				0.296
Never smoking	2,790 (74.9)	407 (76.4)	2,383 (74.7)	
Ever smoking	171 (4.6)	27 (5.1)	144 (4.5)	
Current smoking	764 (20.5)	99 (18.6)	665 (20.8)	
Alcohol consumption, *n* (%)				0.290
Never drinking	3,143 (84.4)	456 (85.6)	2,687 (84.2)	
Ever drinking	46 (1.2)	10 (1.9)	36 (1.1)	
Current drinking	536 (14.4)	67 (12.6)	469 (14.7)	

*SBP, systolic blood pressure; DBP, diastolic blood pressure; BMI, body mass index*.

### Sex-Related Differences in DM Prevalence, Awareness, Treatment, and Control, by Age

Table 2 shows a significantly higher prevalence of DM among older individuals and among women (compared with men, *P* < 0.001). Sex-related differences in DM prevalence were found among those aged 45–54 years and 65–74 years, with a male preponderance among the 45–54-year-olds and a female preponderance among the 65–74-year-olds. DM awareness was greater among women (56.5%) than among men (44.9%, *P* = 0.009), overall. However, poor DM control was more frequently observed among women aged 55–64 years (13.1%) than among similarly aged men (31.0%).

**Table 2:** Sex differences in the prevalence, awareness, treatment, and control rates of DM by age groups.

**Category**	**Total**	**Men**	**Women**	***P***
**PREVALENCE RATE**, ***n*** **(%)**				
45~54 years	129 (10.7)	63 (14.9)	66 (8.5)	0.001
55~64 years	218 (14.6)	81 (13.0)	137 (15.7)	0.149
65~74 years	136 (19.0)	49 (14.8)	87 (22.7)	0.007
≥75 years	50 (16.1)	23 (14.6)	27 (17.8)	0.443
Total	533 (14.3)	216 (14.1)	317 (14.5)	0.719
**AWARENESS RATE**, ***n*** **(%)**				
45~54 years	68 (52.7)	28 (44.4)	40 (60.6)	0.066
55~64 years	122 (56.0)	39 (48.1)	83 (39.4)	0.074
65~74 years	69 (50.7)	23 (46.9)	46 (52.9)	0.506
≥75 years	17 (34.0)	7 (30.4)	10 (37.0)	0.623
Total	276 (51.8)	97 (44.9)	179 (56.5)	0.009
**TREATMENT RATE**, ***n*** **(%)**				
45~54 years	49 (38.0)	21 (33.3)	28 (42.4)	0.288
55~64 years	90 (41.3)	29 (35.8)	61 (44.5)	0.206
65~74 years	56 (41.2)	20 (40.8)	36 (41.4)	0.949
≥75 years	11 (22.0)	5 (21.7)	6 (22.2)	0.967
Total	206 (38.6)	75 (34.7)	131 (41.3)	0.124
**CONTROL RATE**, ***n*** **(%)**				
45~54 years	5 (10.2)	1 (4.8)	4 (14.3)	0.276
55~64 years	17 (18.9)	9 (31.0)	8 (13.1)	0.042
65~74 years	5 (8.9)	3 (15.0)	2 (5.6)	0.235
≥75 years	2 (18.2)	1 (20.0)	1 (16.7)	0.887
Total	29 (14.1)	14 (18.7)	15 (11.5)	0.152

### Sex-Related Differences in DM Prevalence, Awareness, Treatment, and Control, by Education Level

There was an inverse association of DM prevalence with education level, overall, and among women; DM prevalence decreased with an increasing level of education (both, *P* < 0.001). A sex-related difference in the prevalence of DM was found in the illiterate group; the prevalence of DM was higher among women (19.2%) than among men (10.4%, *P* = 0.017). Among those with 1–6 years of formal education, men were more likely to exhibit DM control (28.6%) than women were (11.1, *P* = 0.036). However, men with >6 years of education had a worse awareness rate (36.5%) than did women within the same educational level (50.7%, *P* = 0.017) (Table 3).

**Table 3:** Sex differences in the prevalence, awareness, treatment, and control rates of DM by education level groups.

**Category**	**Total**	**Men**	**Women**	***P***
**PREVALENCE RATE**, ***n*** **(%)**				
0 years	113 (17.4)	14 (10.4)	99 (19.2)	0.017
1~6 years	253 (15.2)	106 (15.3)	147 (15.1)	0.883
> 6 years	167 (11.9)	96 (13.5)	71 (10.2)	0.053
**AWARENESS RATE**, ***n*** **(%)**				
0 years	56 (49.6)	6 (42.9)	50 (50.5)	0.592
1~6 years	132 (52.2)	48 (45.3)	84 (57.1)	0.062
> 6 years	88 (52.7)	43 (44.8)	45 (63.4)	0.017
**TREATMENT RATE**, ***n*** **(%)**				
0 years	46 (40.7)	5 (35.7)	41 (41.4)	0.685
1~6 years	89 (35.2)	35 (33.0)	54 (36.7)	0.541
> 6 years	71 (42.5)	35 (36.5)	36 (50.7)	0.066
**CONTROL RATE**, ***n*** **(%)**				
0 years	5 (10.9)	0	5 (12.2)	1.000
1~6 years	16 (18.0)	10 (28.6)	6 (11.1)	0.036
> 6 years	8 (11.3)	4 (11.4)	4 (11.1)	1.000

### Sex-Related Differences in DM Prevalence, Awareness, Treatment, and Control, by BMI

Table 4 shows that DM prevalence increased with increasing BMI among all individuals, regardless of sex; the prevalence was highest among obese individuals. However, a sex-related difference was observed in the overweight group, with higher rates of awareness and treatment among women (64.7 and 46.7%, respectively) than among men (42.7 and 30.2%, respectively).

**Table 4:** Sex differences in the prevalence, awareness, treatment, and control rates of DM by BMI groups.

**Category**	**Total**	**Men**	**Women**	***P***
**PREVALENCE RATE**, ***n*** **(%)**				
Normal weight	111 (9.2)	46 (8.0)	95 (9.3)	0.389
Over-weight	246 (15.6)	96 (15.1)	150 (16.0)	0.602
Obesity	176 (20.0)	74 (23.1)	102 (18.3)	0.091
**AWARENESS RATE**, ***n*** **(%)**				
Normal weight	58 (52.3)	24 (52.2)	34 (52.3)	0.989
Over-weight	138 (56.1)	41 (42.7)	97 (64.7)	0.001
Obesity	80 (45.5)	32 (43.2)	48 (47.1)	0.616
**TREATMENT RATE**, ***n*** **(%)**				
Normal weight	43 (38.7)	19 (41.3)	24 (36.9)	0.641
Over-weight	99 (40.2)	29 (30.2)	70 (46.7)	0.010
Obesity	64 (36.4)	27 (36.5)	37 (36.3)	0.977
**CONTROL RATE**, ***n*** **(%)**				
Normal weight	10 (23.3)	6 (31.6)	4 (16.7)	0.295
Over-weight	11 (11.1)	5 (17.2)	6 (8.6)	0.212
Obesity	8 (12.5)	3 (11.1)	5 (13.5)	1.000

### Sex-Related Differences in DM Prevalence, Awareness, Treatment, and Control, by BP

With increasing BP measurements, DM prevalence increased significantly among all participants (Table 5).

**Table 5:** Sex differences in the prevalence, awareness, treatment, and control rates of DM by blood pressure level.

**Category**	**Total**	**Men**	**Women**	***P***
**PREVALENCE RATE**, ***n*** **(%)**				
Normal	88 (7.5)	34 (7.7)	54 (7.4)	0.832
Stage I	198 (15.7)	89 (16.3)	109 (15.2)	0.571
Stage II	164 (19.7)	62 (17.1)	102 (21.7)	0.097
Stage III	49 (18.6)	20 (16.1)	29 (20.9)	0.325
**AWARENESS RATE**, ***n*** **(%)**				
Normal	52 (59.1)	15 (44.1)	37 (68.5)	0.023
Stage I	95 (48.0)	36 (40.4)	59 (54.1)	0.055
Stage II	83 (50.6)	28 (45.2)	55 (53.9)	0.277
Stage III	25 (51.0)	11 (55.0)	14 (48.3)	0.644
**TREATMENT RATE**, ***n*** **(%)**				
Normal	40 (45.5)	12 (35.3)	28 (51.9)	0.129
Stage I	71 (35.9)	27 (30.3)	44 (40.4)	0.143
Stage II	59 (36.0)	22 (35.5)	37 (36.3)	0.919
Stage III	19 (38.8)	8 (40.0)	11 (37.9)	0.884
**CONTROL RATE**, ***n*** **(%)**				
Normal	8 (20.0)	1 (8.3)	7 (25.0)	0.396
Stage I	10 (14.1)	6 (22.2)	4 (9.1)	0.164
Stage II	8 (13.6)	6 (27.3)	2 (5.4)	0.043
Stage III	0	0	0	—

Moreover, there was a higher awareness rate among normotensive women than among normotensive men; a worse control rate was observed among women (5.4%) with stage II hypertension than among similarly diagnosed men (27.3%, *P* = 0.043).

## Discussion

This study is the first to explore sex-related differences in the prevalence, awareness, treatment, and control of DM among adults, >45-years-old, in rural areas of northern China. The overall DM prevalence and rates of awareness, treatment, and control were 14.3, 51.8, 38.6, and 14.1%, respectively. Overall, DM awareness was higher among women than among men. DM prevalence increased with increasing age but decreased with increasing levels of education among women; disease prevalence also increased with BMI and BP level, regardless of sex. A male predominance in DM prevalence was observed only for individuals 45–54-years-old; a female predominance presented among individuals aged 65–74 years and among those who were illiterate. A sex-related difference in awareness rates was observed among those with >6 years of education, those who were overweight. Among those with normal BP, higher rates were observed in women than among men. There also appeared to be higher treatment rates among overweight women, compared with overweight men. Among participants aged 55–64 years, those with 1–6 years of education, and those with stage II hypertension, men demonstrated higher control rates than women did.

A 2017 IDF report showed that 425 million people, worldwide, have DM, yielding a prevalence of 8.6% (28). The prevalence of DM among elderly Americans is 21%, with awareness, treatment, and control rates of 71, 51, and 50%, respectively (15). In 2010, the overall DM prevalence and rates of awareness, treatment, and control were estimated to be 11.6, 30.1, 25.8, and 39.7%, respectively, among adults in China (14). Compared with previous studies, the present study showed a higher prevalence of DM (14.3%) among adults ≥45-years-old; however, our observed rates of awareness (51.8%), treatment (38.6%), and control (4.1%) were disproportionately low. These data suggest that diabetes may have reached alert levels in this study population and, without effective intervention, diabetes-related complications (including cardiovascular disease, stroke, and chronic kidney disease) may be prevalent in the future.

The prevalence of DM increased with age (29, 30) and was strongly associated with education level, BMI, and hypertension (31–36). The sex-related difference in the association of DM with age was identified in a previous study. There were differences in the prevalence of DM between men and women at different ages; the disease was more prevalent among men < 50-years-old and among women >60-years-old (14). Older age was identified as a stronger risk factor for developing DM among women than among men (37). In this study, men exhibited a higher prevalence of DM than women among the 45–54-year-olds; however, women had a higher prevalence than men among the 65–74-year-olds.

In a previous bivariate analysis, those with DM were less well-educated than those without the disease (36). Women who had not completed at least 8 years of formal education had a 1.45-times greater risk of developing DM than more educated women did; a similar trend was not observed among men (31). Consistent with previous studies, there was a significant inverse relationship between education level and DM prevalence among women; among the illiterate participants, women demonstrated a higher prevalence of DM than men did. This finding supports the need for strengthening rural education, especially among women; education is associated with several health benefits.

In the United States and Kazakhstan, women with DM are more likely than men to be aware of their condition (15, 38). One study showed that awareness increases significantly with higher levels of education (39), as suggested by another study (17). A study from Switzerland showed that high BMIs were also positively correlated with type 2 DM awareness (40). In the present study, DM awareness was higher among women than among men overall, as well as for those with >6 years of formal education, those who were overweight, and those who were normotensive. The results also showed that women were more concerned about their health status than men were, in this study population.

Male sex and increasing age were significantly and positively associated with antidiabetic drug treatment in Switzerland (40). Among Chinese adults, in 2010, only 25.8% of the overall population (men, 25.5%; women, 26.2%) with DM was actually treated for this condition (14). Contrary to some previous studies, the present study did not reveal any differences between men and women in terms of DM treatment rates, overall. However, for those who were overweight, the DM treatment rate was higher among women than among men. Obesity/bodyweight gain is the strongest modifiable risk factor for DM (41–43). Hence, bodyweight control is potentially the simplest and one of the most effective strategies for preventing DM, especially among men.

DM control rates were substantially higher among women (31.8%) than among men (22.6%) in older individuals in Kazakhstan (38). Among Chinese adults receiving DM treatment, 39.7% demonstrated good blood glucose control (glycated hemoglobin levels <7.0%); 40.7% in men and 38.6% in women (14). Previous studies have shown a statistically significant association between age and DM control rates among women but not among men, and no association has been demonstrated between the diabetes control rate and education level (17). However, in the present study, men demonstrated higher control rates than women among the 55–64-year-olds, those with 1–6 years of education, and among individuals with stage II hypertension. These observations may be related to the benefits of the health education movement and/or the sociocultural focus on improving health status. Men aged 55–64 years remain the main workforce of the family, so families are investing more in the treatment of diseases in this segment of the population.

This study had several limitations. First, the study population was recruited from villages in Tianjin, China, so the findings may not be generalizable to the whole Chinese population. Second, the cross-sectional study design may have led to selection bias, with a response rate of 75%. Third, only FPG levels, as indicators of diabetes, were measured in all participants; the lack of impaired glucose tolerance testing and glycated hemoglobin level determinations may have underestimated the prevalence of DM. Moreover, reliance on self-reporting of DM, in this poorly educated population, may have also contributed to an underestimation of the number of individuals with DM.

## Conclusion

This is the first report regarding sex-related differences in DM prevalence and its rates of awareness, treatment, and control among adults ≥45-years-old in rural areas of northern China. The DM prevalence and its rates of awareness, treatment, and control were 14.3, 51.8, 38.6, and 14.1%, respectively. Among the younger segment of the study population, men had a higher prevalence of DM than women, but the inverse was observed among the elderly. Middle-aged men and those with stage II hypertension men had better control of their FPG levels than the corresponding women category. However, women were more likely to be aware of their DM than men, especially among overweight women and those with normal BP. The results of this study suggest that DM awareness should be improved among men; younger men, especially, should be regularly screened for DM. In addition, DM education and management should be encouraged for elderly women, especially those with low education levels. Further studies are necessary to explore this situation among representative population samples in China in order to establish effective protocols against DM.

## Author Contributions

JW and XN were involved in conception, design, and data collection. JLi and JN were involved in manuscript drafting for this article. JLi, JN, YW, HZ, JLiu, JT, JC, XN, and JW were involved in data collection and case diagnosis and confirmation for this article. JW and XN were involved in data analysis, and data interpretation. JW, XN, and JC were involved critical review for this article. All authors read and approved the final manuscript.

### Conflict of Interest Statement

The authors declare that the research was conducted in the absence of any commercial or financial relationships that could be construed as a potential conflict of interest.
